# Evaluation of the Combination of Metformin and Rapamycin in an MPP^+^-Treated SH-SY5Y Model of Parkinson's Disease

**DOI:** 10.1155/2023/3830861

**Published:** 2023-01-16

**Authors:** Chureerat Norradee, Kawinthra Khwanraj, Tatcha Balit, Permphan Dharmasaroja

**Affiliations:** ^1^Department of Anatomy, Faculty of Science, Mahidol University, Bangkok, Thailand; ^2^Chakri Naruebodindra Medical Institute, Faculty of Medicine Ramathibodi Hospital, Mahidol University, Samut Prakan, Thailand

## Abstract

Metformin (MET) and rapamycin (RAPA) have been reported to protect against neurodegeneration in cellular and animal models of Parkinson's disease (PD). MET, which is a first-line drug for type 2 diabetes, and RAPA are known as mTORC1 inhibitors. MET also acts as an AMPK activator, which leads to the inhibition of mTORC1 activity. mTORC1 is a downstream target of Akt signaling. Inactivation of Akt/mTORC1 and its downstream S6K1 can promote autophagy, a process involved in PD pathogenesis. Based on their mechanisms and potential benefits, we evaluated the potential protective effect of pretreatment with combinations of MET and RAPA in a 1-methyl-4-phenylpyridinium ion (MPP^+^)-treated SH-SY5Y neuronal cell model of PD. The results showed that MET and RAPA combinations lowered cell viability after exposure to MPP^+^. Increased LC3-II levels by MPP^+^ were not altered by MET and RAPA pretreatment. In normal neuronal cells, MET and RAPA pretreatment inhibited the phosphorylation of both Akt and S6K1, and the phosphorylation remained suppressed after MPP^+^ exposure. These findings suggest that when cells were exposed to MPP^+^, suppressed phosphorylation of both Akt and S6K1 by the MET and RAPA combination may lead to an inappropriate autophagic response, resulting in increased cell death.

## 1. Introduction

Metformin (MET; dimethylbiguanide) is widely used as the first-line treatment of type 2 diabetes mellitus (T2DM). It has been reported that MET acts as an AMPK activator and mTORC1 inhibitor [1, 2]. AMPK is upstream of mTORC1, and its activation inhibits mTORC1 activity. The potential roles of MET in neurodegenerative diseases have been studied. Pretreatment of MET protected against neurotoxicity in 1-methyl-4-phenylpyridinium ion (MPP^+^) cellular model of Parkinson's disease (PD) [3]. The mechanism of MET in neuroprotection remains unclear. A study in breast cancer cells showed that MET decreases Akt activation and promotes apoptotic death [4], while in SH-SY5Y neuroblastoma cells, phosphorylated Akt was increased by MET treatment, which is associated with neuronal differentiation [5].

Rapamycin (RAPA) and its analogs are known as a potent mTORC1 inhibitor. Inhibition of mTORC1 by RAPA induces alpha-synuclein autophagy and reduces neurodegeneration in the 1-methyl-4-phenyl-1,2,3,6-tetrahydropyridine (MPTP) mouse model of PD [6]. A combination of MET and RAPA was found to inhibit the growth and progression of prostate tumors, with a significant reduction in mTORC1 signaling [7]. Whether blocking of mTORC1 signaling by a combination of MET and RAPA can protect against neuronal death in PD remains to be investigated.

mTORC1 is a downstream target of Akt signaling. In a 6-hydroxydopamine (6-OHDA) cellular model of PD, activation of Akt inhibits autophagy, which can be mediated by activation of mTOR [8]. It is known that the mTORC1-S6K1 pathway regulates autophagy by phosphorylation and inactivation of proteins involved in autophagosome formation [9]. Inhibition of S6K1 increases the conversion of microtubule-associated protein light chain 3 (LC3) from LC3-I to LC3-II, a marker for autophagosomes, which is mediated through AMPK activation [10]. While MET activates AMPK, it has been shown that RAPA inhibits the AMPK-mTOR signaling pathway in hippocampal neurons of T2DM rats [11]. In addition, previous studies have shown that RAPA activates Akt signaling via a negative feedback loop while inhibiting mTORC1 signaling [12]. While MET and RAPA have a similar effect on the inhibition of mTORC1, which may be beneficial for their combination on neuroprotection in PD, the outcome of the combination could be affected by their distinct effects on AMPK and Akt.

Thus, we evaluated the potential protective effect of pretreatment with a combination of MET and RAPA in SH-SY5Y neuronal cells after exposure to MPP^+^ and investigated the expression of LC3-II, and activation of Akt and S6K1.

## 2. Materials and Methods

### 2.1. Cell Culture and Differentiation

SH-SY5Y neuroblastoma cells were grown in 1 : 1 mixture of minimum essential media and Ham' s F12 nutrient mixture (Gibco, New York, USA), supplemented with 10% heat-inactivated fetal bovine serum (FBS; Hyclone, Gaithersburg, Missouri, USA) and maintained at 37°C under humidified 5% CO_2_ atmosphere. Neuronal differentiation was induced with 10 *μ*M all-trans retinoic acid (RA; Sigma-Aldrich, MO, USA) in a medium supplemented with 1% FBS for 10 days. During RA treatment, the media was replaced with 10 *μ*M RA in fresh medium supplemented with 1% FBS every 2 days. Cell morphology was visualized using an inverted epifluorescence microscope.

### 2.2. Immunocytochemistry for TH and MAP2 Expression

Cells were seeded at an initial concentration of 5 × 10^4^ cells/well and were grown on poly-L-lysine coated coverslips in 24-well plates. Cells were fixed for 15 minutes with 4% paraformaldehyde at room temperature and permeabilized with 0.25% Triton *X*-100 in PBS. Cells were then washed with PBS and incubated in a blocking solution (1% BSA in 0.5% Tween 20 in PBS) for 30 minutes followed by overnight incubation at 4°C with the rabbit antibody against tyrosine hydroxylase (TH; Cell Signaling, Massachusetts, USA) or the rabbit antibody against microtubule-associated protein 2 (MAP2; Cell Signaling). After washing with PBS, cells were incubated with fluorescent dye Alexa 488-conjugated or Alexa 594-conjugated goat antirabbit IgG secondary antibody (Cell Signaling) for 2 h at room temperature. Nuclei were counterstained with Hoechst 33342 (Abcam, Massachusetts, USA). Immunostaining was visualized under a fluorescence microscope (Olympus BX53, Tokyo, Japan).

### 2.3. Treatment with MPP^+^, Metformin, and Rapamycin

To evaluate an appropriate concentration of 1-methyl-4-phenylpyridinium ion (MPP^+^; Sigma‐Aldrich), differentiated SH-SY5Y cells were treated with 700 and 1000 *μ*M MPP^+^ for 24 hours. To investigate the effect of MET (Sigma‐Aldrich) on MPP^+^-treated cells, cells were pretreated with 0.5 and 1 mM MET for 24 hours before exposure to MPP^+^. To investigate the effect of RAPA (Thermo Fisher Scientific, MA, USA) on MPP^+^-treated cells, cells were pretreated with 0.5, 2, and 10 *μ*M RAPA for 24 h before exposure to MPP^+^.

### 2.4. Measurement of Cell Viability

Cell viability was determined using a 3‐[4, 5‐dimethylthiazol‐2‐yl]‐2, 5‐diphenyltetrazolium bromide (MTT; Sigma-Aldrich) assay. Cells were seeded in 96‐well plates at a density of 5 × 10^4^ cells/well. After treatment, MTT was added to each well at a final concentration of 0.5 mg/mL before incubation for 3 hours. Then, 100 *μ*M DMSO was added to dissolve formazan crystals for 15 minutes. Absorbance at 570 and 690 nm was measured by a microplate reader (BioTek, Shanghai, China). The results were represented as a percentage of the control group value.

### 2.5. Western Blotting Analysis of TH, LC3, Akt, and S6K1 Expression

After treatment, cells were washed with PBS, trypsinized, and centrifuged. Cells were lysed in lysis buffer containing a 1% protease inhibitor cocktail (Sigma‐Aldrich) and incubated on ice, followed by centrifugation at 14,000 rpm for 20 minutes. The supernatant was collected and the protein concentrations were determined using the BCA Protein Assay Kit (Thermo Fisher Scientific). Protein samples were separated on a 4–15% SDS-PAGE and transferred onto a PVDF membrane. After blocking with 5% skim milk for 2 hours, membranes were incubated with the following primary antibodies at 4°C overnight: rabbit anti-LC3 (1 : 1000; Sigma-Aldrich), rabbit antiAkt (1 : 1000; Cell Signaling), rabbit antiphospho-Akt (Ser473; 1 : 1000; Cell Signaling), rabbit anti-p70S6K1 (1 : 1000; Cell Signaling), rabbit antiphospho-p70S6K1 (Thr421/Ser424; 1 : 1000; Cell Signaling), and mouse anti-*β*-actin (1 : 5000; Abcam). For TH, undifferentiated and differentiated cells were incubated with rabbit anti-TH (1 : 1000; Cell Signaling). Next, membranes were washed in TBS-T buffer and incubated with HRP conjugated antirabbit (1 : 10000; Cell Signaling) or HRP conjugated antimouse IgG (1 : 10000; Cell Signaling) in blocking buffer for 2 hours at room temperature. Immunoreactive proteins were detected using the ECL chemiluminescence system (UVITEC). The immunoblot density was quantitated using ImageJ software (National Institute of Health, MD, USA).

### 2.6. Statistical Analysis

Results were expressed as mean ± standard error of the mean (SEM). Mean comparisons between the two samples were determined by Student's *t*-test. For multiple comparisons, data were analyzed using a one-way analysis of variance (ANOVA) followed by a post-hoc analysis (Tukey's multiple comparison test) using GraphPad Prism 5 software. A value of *p* < 0.05 was considered statistically significant.

## 3. Results

### 3.1. Differentiation of SH-SY5Y Cells into a Neuronal Phenotype

**Figure 1:**
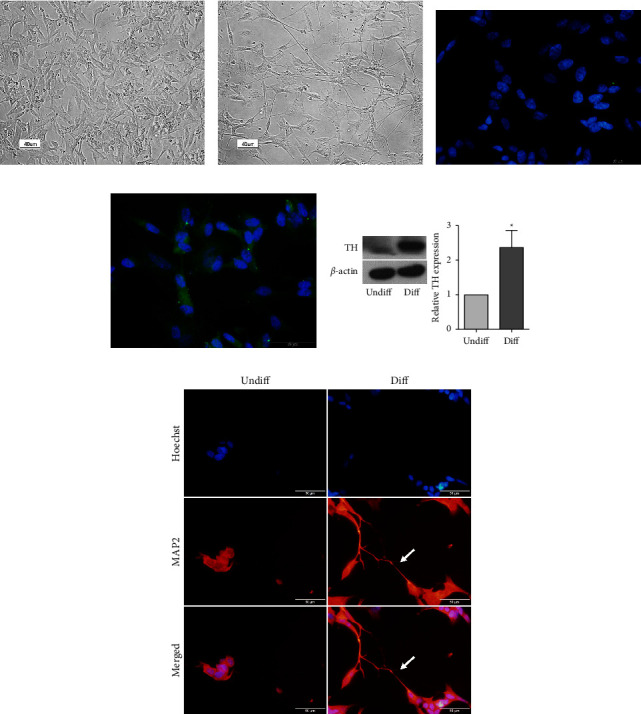
Differentiation of SH-SY5Y cells into a neuronal phenotype. SH-SY5Y cells were treated with 10 *μ*M RA for 10 days for neuronal differentiation. Inverted epifluorescence microscope revealed morphology of (a) undifferentiated and (b) differentiated cells. Scale bar = 40 *μ*m. Expression of TH in (c) undifferentiated and (d) differentiated cells was visualized using immunofluorescence staining: Hoechst nuclear staining in blue and TH in green. Scale bar = 50 *μ*m. (e) Western blotting analysis and quantification of TH in undifferentiated and differentiated cells expressed as mean ± SEM (*n* = 3). ^*∗*^*p* < 0.05 by student's *t*-test. (f) Expression of MAP2 in 5-day differentiated cells using immunofluorescence staining: Hoechst nuclear staining in blue and MAP2 in red. Scale bar = 40 *μ*m. White arrows indicate neurite outgrowth.

To evaluate the effects of MET and RAPA in neuronal cells, SH-SY5Y cells were treated with 10 *μ*M RA to induce neuronal differentiation. After 10 days of induction, SH-SY5Y cells showed marked neuritic extension (Figure 1(b)), compared with undifferentiated cells (Figure 1(a)). Immunofluorescence showed a higher expression of TH, the rate-limiting enzyme of dopamine biosynthesis, in differentiated cells (Figure 1(d)) than in undifferentiated cells (Figure 1(c)). The protein expression of TH was confirmed by Western blot analysis showing a significantly increased TH expression in differentiated neuronal cells (Figure 1(e)). This differentiation protocol was used to generate TH-expressing neuronal cells for subsequent experiments. Neuronal differentiation was also confirmed by MAP2 immunofluorescence staining showing the extension of neurites in 5-day differentiated SH-SY5Y cells (Figure 1(f)).

### 3.2. Determining the MPP^+^ Concentration for Generating a PD Model

**Figure 2:**
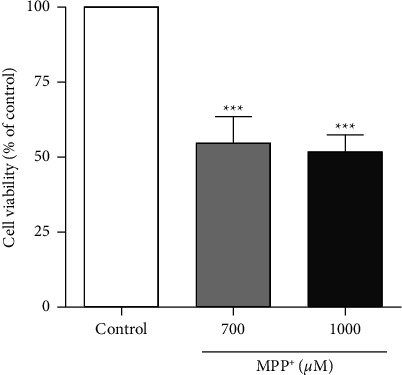
Effects of MPP^+^ on cell viability in differentiated SH-SY5Y cells. Differentiated SH-SY5Y cells were treated with MPP^+^ at 700 and 1000 *μ*M for 24 hours. After treatment, cell viability was measured by using MTT assay. Values represent mean ± SEM (*n* = 3 with five replicates each). ^*∗∗∗*^*p* < 0.001 compared with the control group.

To generate a PD cellular model, differentiated SH-SY5Y cells were treated with MPP^+^, a potent complex I inhibitor in dopamine neurons, at 700 *μ*M and 1000 *μ*M for 24 hours, and cell survival was assessed using an MTT assay. When compared with the untreated control, 700 *μ*M and 1000 *μ*M MPP^+^ significantly reduced cell viability to 54.7% and 51.8%, respectively, (Figure 2). Thus, 700 *μ*M of MPP^+^ was used to induce neuronal cell death in subsequent experiments.

### 3.3. Effects of MET and RAPA Combination on the Survival of MPP^+^-Treated Neuronal Cells

**Figure 3:**
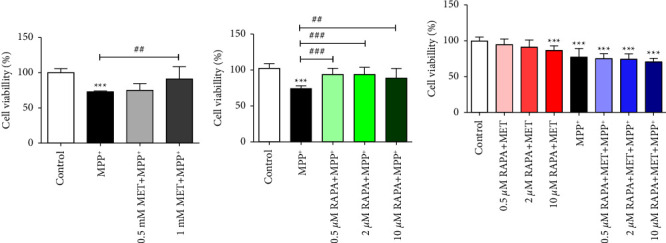
Effects of pretreatment of MET, RAPA, and MET and RAPA combinations on cell viability of MPP^+^-treated differentiated SH-SY5Y cells. Cell viability was assessed using an MTT assay. After pretreatment of (a) MET, (b) RAPA, and (c) MET and RAPA combinations for 24 hours, cells were exposed to 700 *μ*M MPP^+^ for 24 hours. Values represent mean ± SEM (*n* = 3 with triplicates each). ^*∗∗∗*^*p* < 0.001 compared with the control group. ^##^*p* < 0.01, ^###^*p* < 0.001 compared with the MPP^+^ group.

As mentioned earlier, studies have suggested that MET or RAPA protects against neurotoxicity in cellular and animal models of PD. Here, we started with an MTT assay to assess the effect of 24-hour exposure of MET or RAPA on SH-SY5Y cells that were induced by RA for 10 days to differentiate into a neuronal phenotype prior to exposure to 700 *μ*M MPP^+^ for 24 hours. The results showed that 1 mM MET could protect against the loss of viability of the cells (*p* < 0.01; Figure 3(a)). Similar effects were seen for RAPA at 0.5, 2, and 10 *μ*M (Figure 3(b)). However, the combination of 1 mM MET with various concentrations of RAPA did not protect neuronal cells against MPP^+^-induced cell death (Figure 3(c)). The results also revealed that the combination of MET with a higher concentration of RAPA at 10 *μ*M increased cell death in MPP^+^-untreated neuronal cells (*p* < 0.001).

### 3.4. Effects of MET and RAPA Combinations on LC3 Conversion

**Figure 4:**
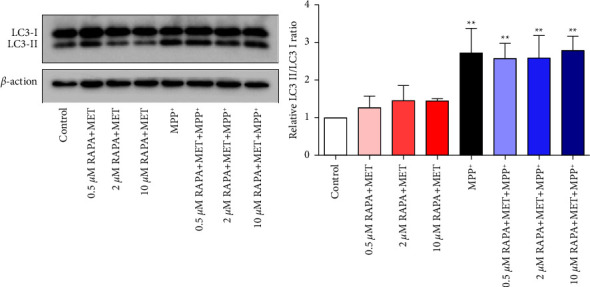
Western blotting analysis of LC3 expression in MPP^+^-treated differentiated SH-SY5Y cells pretreated with MET and RAPA combinations. After pretreatment for 24 hours, cells were exposed to 700 *μ*M MPP^+^ for 24 hours. Quantification of the band density was normalized to *β*-actin. Values represent mean ± SEM. (*n* = 3). ^*∗∗*^*p* < 0.01 compared with the control group.

To evaluate whether increased death in neuronal cells treated with MET and RAPA combinations and MPP^+^ exposure was associated with the magnitude of autophagy, we determined cellular LC3-I and LC3-II levels by performing Western blotting analysis. Treatment with MET and RAPA combinations did not affect basal levels of LC3; however, after MPP^+^ exposure, LC3 conversion was increased, and the LC3-II/LC3-I ratios were at a similar level observed in cells that received MPP^+^ alone (Figure 4). MPP^+^ alone significantly increased LC3-II expression (*p* < 0.01), which was also seen in our previous study [13]. Although activation of autophagy prevented neurodegeneration in an MPTP mouse model of PD [6], excessive activation of this pathway has also been associated with cell death.

### 3.5. Effects of MET and RAPA Combination on Akt and S6K1 Phosphorylation

**Figure 5:**
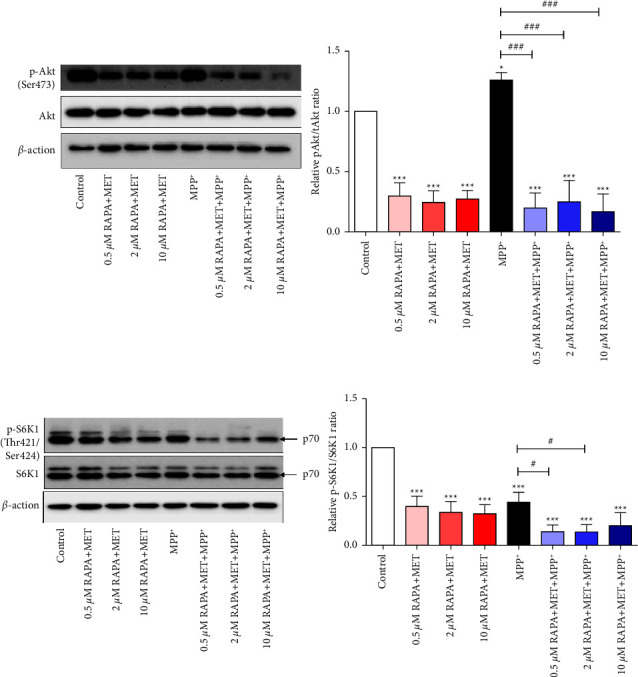
Western blotting analysis of Akt and S6K1 expression in MPP^+^-treated differentiated SH-SY5Y cells pretreated with MET and RAPA combinations. After pretreatment, cells were exposed to 700 *μ*M MPP^+^ for 24 hours. (a) Representative blots of Akt and phospho-Akt and quantification of relative ratio of phospho-Akt to total Akt. (b) Representative blots of S6K1 and phospho-S6K1 and quantification of relative ratio of phospho-A6K1 to total S6K1. The band density was normalized to *β*-actin. Values represent mean ± SEM (*n* = 3). ^*∗*^*p* < 0.05. ^*∗∗∗*^*p* < 0.001 compared with the control group. ^#^*p* < 0.05. ^###^*p* < 0.001 compared with the MPP^+^ group.

It is known that activation of the Akt/mTORC1/S6K1 pathway regulates autophagy [8, 9]. We determined the phosphorylation of Akt and S6K1 using Western blotting analysis. The results showed that MET and RAPA combinations markedly suppressed the phosphorylation of Akt in both normal neuronal cells and cells after MPP^+^ exposure (Figure 5(a)). MPP^+^ alone increased Akt phosphorylation. The findings suggested that the combination of MET and RAPA suppressed Akt activation at the basal level, rendering the cells unable to respond to MPP^+^. MET and RAPA combination also significantly suppressed the phosphorylation of S6K1 in both normal neuronal cells and cells after MPP^+^ exposure (Figure 5(b)). Although S6K1 is a downstream molecule of Akt, it exerts negative feedback on Akt [14]. As expected, MPP^+^ alone decreased S6K1 phosphorylation, which is consistent with a previous study in a PD in vitro model [15]. Overall, the results suggested that the combination of MET and RAPA suppressed the phosphorylation of both Akt and S6K1, not only in physiologically normal cells but also in cells with MPP^+^-induced toxicity.

## 4. Discussion

MET and RAPA have been investigated as a potential neuroprotective agent in PD. Increasing evidence suggests that dysregulation of the autophagy process in the accumulation of alpha-synuclein and/or damaged mitochondria, which is commonly observed in PD [6, 13]. In the process of autophagy, the cytoplasmic contents are degraded inside the lysosome/vacuole and the resulting macromolecular components are recycled. Macroautophagy, henceforth-called autophagy, is one type of autophagy process that involves the sequestration of substrates within cytosolic double-membrane vesicles called autophagosomes. After degrading, the breakdown products are returned to the cytosol to recycle the macromolecular components, provide energy to sustain cell viability in unfavorable settings, and protect the cell under various conditions of stress [16]. MET is widely known as an AMPK activator that promotes AMPK phosphorylation and induces autophagy. AMPK is upstream of mTORC1, and the activation of AMPK inhibits mTORC1 activity. Inhibition of mTORC1 by RAPA induces autophagy in the MPTP model of PD [6]. Thus, we hypothesized that the MET and RAPA combination might provide more neuroprotective effects in PD.

In our study, MPP^+^-treated SH-SY5Y neuronal cells were used as a PD model to evaluate the effects of MET and RAPA combinations on cell viability and an autophagy marker, LC3-II, including the autophagy-related Akt/S6K1 pathway. The results showed that pretreatment of combinations of 1 mM MET and 0.5, 2, and 10 *μ*M RAPA before MPP^+^ exposure did not protect against cell death, compared with the MPP^+^ alone group. In addition, increased LC3-II levels by MPP^+^ were not altered by MET and RAPA combination. It is plausible that the drug combination may influence the basal levels of autophagy. Increasing evidence supports that dysregulated or inadequate autophagy is closely related to PD pathogenesis [6, 8, 13]. However, it cannot be ruled out that MPP^+^ exposure after MET and RAPA treatment may promote excessive activation of the autophagic pathway, resulting in increased neuronal death, as shown in Figure 3(c). A previous study in cultured skeletal myotubes supports our findings, in physiologically normal neuronal cells, that the ratio of LC3-II/I was not increased by MET and RAPA combination, and the combination lowered mitochondrial protein synthesis rates and slowed cellular proliferation, which may explain increased cell death after MPP^+^ exposure in our study [17].

**Figure 6:**
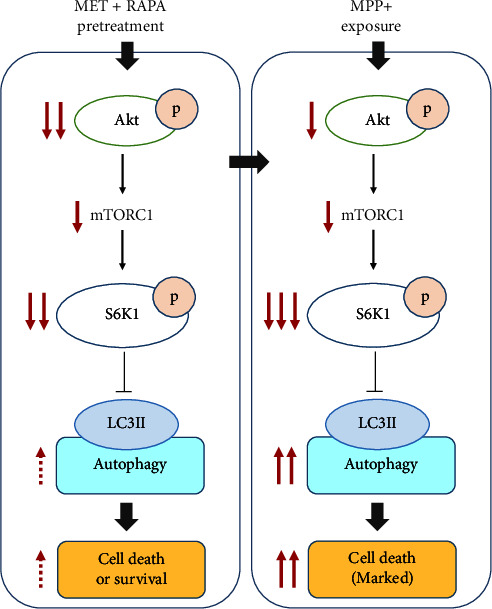
Schematic of the Akt/S6K1 pathway in the modulation of autophagy in MPP^+^-treated neuronal cells pretreated with MET and RAPA combination. SH-SY5Y cells were treated with 10 *μ*M RA for 10 days for neuronal differentiation before exposure to MET and RAPA. The number of red arrows indicates the strength of the effect.

We assessed the activation of the Akt/S6K1 pathway, which is known to regulate autophagy [8, 9]. A previous study in the dopaminergic neuron MN9D showed that MPP^+^ decreased S6K1 phosphorylation and reduced neuronal cell viability [15]. S6K1 is downstream of Akt, and it has a negative feedback effect on Akt by inhibiting the binding of insulin receptor substrate (IRS) to PI3K [14]. Thus, decreased S6K1 phosphorylation induced by MPP^+^ increases Akt activation, as also seen in our results. In most cellular systems, Akt activation inhibits autophagy; however, increased LC3 conversion by MPP^+^ could be a result of S6K1 inhibition that is mediated through AMPK activation. A previous study demonstrated that suppression of S6K1 activity led to the activation of AMPK, which then phosphorylated ULK1 and induced autophagy [10]. In our study, MET and RAPA pretreatment inhibited both Akt and S6K1 phosphorylation in normal neuronal cells, and the phosphorylation remained suppressed after MPP^+^ exposure. Decreased phosphorylation of both Akt and S6K1 by MET and RAPA combinations may lead to an increase in autophagy when cells were exposed to MPP^+^, as shown by increased LC3-II levels in Figure 4.  Figure 6 summarizes our findings on the Akt/S6K1 pathway in the modulation of autophagy occurring in the neuronal cells. Suppression of the Akt/S6K1 pathway by MET and RAPA pretreatment initiates autophagy, which can function as a survival mechanism or a dying mechanism dependent on the concentration of MET and RAPA. At high concentrations, the drug combination may lead to neuronal death. After exposure to MPP^+^, the activation of Akt that is typically induced by MPP^+^ remains suppressed by MET and RAPA, leading to increasing of autophagy in the cells, and excessive autophagy due to the continued presence of the stress may trigger a death response.

In conclusion, the combination of MET and RAPA may lead to an inappropriate autophagic response and cannot protect neuronal cells against MPP^+^-induced cell death. MET and RAPA combinations may affect mTORC1 inhibition, AMPK activation, and the S6K1 feedback loop in a complicated way. Determining the interactions of multiple signaling proteins induced by MET and RAPA, which influence the level of autophagy, must be considered for further studies.

## Data Availability

The data that support the findings of this study are available from the corresponding author upon reasonable request.
